# Consensus-Based Cooperative Control Based on Pollution Sensing and Traffic Information for Urban Traffic Networks

**DOI:** 10.3390/s17050953

**Published:** 2017-04-26

**Authors:** Antonio Artuñedo, Raúl M. del Toro, Rodolfo E. Haber

**Affiliations:** Centre for Automation and Robotics (CSIC—UPM), Ctra. Campo Real Km. 0.2, Arganda del Rey, 28500 Madrid, Spain; raul.deltoro@car.upm-csic.es (R.M.d.T.); rodolfo.haber@car.upm-csic.es (R.E.H.)

**Keywords:** air pollution monitoring, cooperative systems, discrete event systems, traffic sensing, urban traffic network

## Abstract

Nowadays many studies are being conducted to develop solutions for improving the performance of urban traffic networks. One of the main challenges is the necessary cooperation among different entities such as vehicles or infrastructure systems and how to exploit the information available through networks of sensors deployed as infrastructures for smart cities. In this work an algorithm for cooperative control of urban subsystems is proposed to provide a solution for mobility problems in cities. The interconnected traffic lights controller (*TLC*) network adapts traffic lights cycles, based on traffic and air pollution sensory information, in order to improve the performance of urban traffic networks. The presence of air pollution in cities is not only caused by road traffic but there are other pollution sources that contribute to increase or decrease the pollution level. Due to the distributed and heterogeneous nature of the different components involved, a system of systems engineering approach is applied to design a consensus-based control algorithm. The designed control strategy contains a consensus-based component that uses the information shared in the network for reaching a consensus in the state of *TLC* network components. Discrete event systems specification is applied for modelling and simulation. The proposed solution is assessed by simulation studies with very promising results to deal with simultaneous responses to both pollution levels and traffic flows in urban traffic networks.

## 1. Introduction

Nowadays, the challenge of making sustainable and environmentally friendly cities is driving the activities of many stakeholders such as local and national authorities, the private sector, environmental pressure groups, university research institutes, and neighborhood associations. They are jointly promoting system-of-systems-based solutions for smart cities [1,2,3]. From a physical and system engineering standpoint, a city or an urban region can be considered as a physical system composed of several coupled physical subsystems with thousands of networked sensors and actuators (e.g., transportation, energy distribution systems, water supply systems, etc.). Thereby, the overall dynamic performance of the city can be assessed on the basis of the emerging behavior of these coupled physical subsystems having three important features: complexity, uncertainty and heterogeneity. Strict requirements for sustainability, resource availability, and optimality are then imposed. The ability to anticipate and control daily situations (e.g., traffic congestion and high levels of pollution) and unexpected events (e.g., power failures, traffic accidents and exceptional climate conditions) is a key issue to deal with complex and potentially unstable conditions. In addition, many sensors, actuators, communication systems, and computing platforms are available in each subsystem. Therefore, the development of integrated methods based on these sensory data, signal processing strategies and decision-making procedures is a challenge with no straightforward mathematical tractability, and difficult representation and evaluation by traditional modeling and simulation tools. New computing platforms are able to measure representative variables, perceive physical events that may occur in the environment, preprocess the events with embedded computing capabilities and send information to networked and distributed applications that can perform more complex tasks for managing complete urban systems.

Current research on smart cities aims to integrate urban subsystems [3]. These subsystems are usually from different domains or with different timing and bandwidths. Two examples are the pollution measurement systems and the traffic control and monitoring systems. The heterogeneity of the systems imposes new challenges in model-based design approaches. On the other hand, the increasing number of sensors, actuators, communication systems and computing devices already deployed in cities, enable new applications to go beyond particular solutions and cover wider urban systems and scenarios. These challenges have been recently addressed in other application fields by using machine learning techniques, data mining or artificial cognition-based strategies [4,5].

The System-of-Systems (SoS) paradigm emerges from the need to address the complexity of interconnected systems. In this context, systems are independently designed, evolve independently and can operate autonomously [6,7]. Nevertheless, the constituent systems of a SoS have their own individual goals while the whole SoS has global goals. The expected goal of operating sub-systems that work together is a better overall yield of the sum of the systems in comparison with each individual system. Their joint operation provides new functionalities; subsystems are also operationally independent and autonomous, so interactions between them are generally asynchronous and can be represented as discrete-event models [8]. This heterogeneity of the systems brings new challenges to the model-based design approach.

How can cooperative control and sensing systems be designed on the basis of SoS paradigm to provide new sensor-based solutions for smart urban systems?

The design of cooperative control strategies for urban subsystems can be supported on centralized and distributed coordination schemes [9]. In a centralized coordination scheme, it is assumed that each system has the ability to communicate with a central unit or share information via a fully connected network. On the contrary, distributed coordination schemes reduce communication requirements using local neighbor-to-neighbor interactions, thus improving scalability, flexibility, reliability and robustness.

Consensus is a fundamental issue to be considered for designing cooperative control for distributed multi-agents coordination. Some reports on cooperative control of sets of multiple subsystems such as vehicles, robots and rovers are inspired in consensus-based approaches [10]. Nanayakkara et al. [8], suggested a consensus-based control using the cooperative-control paradigm, to increase the expected benefits of the resulting system. These authors proposed to create a set of systems each pursuing its own objectives as well as their common goals, using communications between subsystems.

Consensus algorithms have been widely applied to distributed cooperative systems such as vehicle groups [9,11,12], swarm robots [10], traffic control systems [13,14,15], traffic speed density prediction [16] and wireless-sensor networks [17], among others. In these cases, consensus control enables each individual system or agent to ascertain the global goal and then, based on the state of the other system, to make decision on the best control signal to achieve the corresponding goal.

The problem of traffic optimization in urban environments based on city-pollution information can be addressed by a consensus-based approach between different control agents [18]. Focused on a similar problem (urban-traffic management and control), Castro et al. proposed a multilayer distributed model for predictive control of urban traffic. The control system was composed of local and global layers of controls where each control agent is responsible for traffic lights at one intersection. The system uses the principles of consensus between control agents and behavioral coordination, in order to conciliate local and global control objectives.

In another work reported in [19], the distributed control principle was applied to a network of intersections, with the aim of controlling the traffic at a macroscopic level. Wuthishuwong et al. implemented a discrete time-consensus algorithm, to coordinate the gross traffic density of an intersection and its neighborhoods in the network. The traffic density at each intersection was collected, by using Vehicle-to-Infrastructure (V2I) communication, and the information was distributed to the neighborhood via Infrastructure-to-Infrastructure (I2I) communication link. Each intersection performed as a centralized controller that computed the control signal using both, own and neighborhood information on traffic density. Intersections were considered individual nodes, in order to apply the consensus strategy, and each node shared information on its traffic density and coordination variables.

In this work, the System-of-Systems (SoS) engineering paradigm sets the foundation for developing a cooperative control strategy applied to an urban scenario. For this purpose, the air-pollution information service and the traffic control subsystem are put together, and the control system makes use of available information to modify or adjust traffic-light cycles at intersections. Due to the distributed nature of the main subsystems, a distributed consensus-based control method is developed. Pollution levels and traffic densities are considered inputs of the proposed control strategy based on subsystems cooperation. It should be noted that sources of air pollution in cities are not only coming from vehicle emissions but also from industrial activity, domestic fuel burning, boilers for heating, etc.; which increases the complexity for considering the whole problem. Modeling and simulation are not straightforward according to the complexity of main subsystems, instead both can be powerful tools to address the problem and generate the basic knowledge on this field. The discrete event systems specification (DEVS) is applied for modeling and simulation in this work. The main rationale for using DEVS relies on the suitability of this framework for modeling complex dynamical systems and their interactions [20].

From the best of authors’ knowledge the main contribution of this work is the design and evaluation of a strategy for improving the performance of urban traffic networks in specific regions of a city taking into account traffic and pollution sensory information. A consensus-based cooperative control method is proposed by taking advantage of new developments on technologies and concepts for smart cities. This paper is organized as follows: in Section 2, a brief review of pollution and traffic sensing systems is presented. Section 3 introduces the problem and the proposed solution. Section 3.1 deals with the modeling technique for representing the behavior of the main subsystems components. Section 3.2 addresses the consensus-based cooperative control and presents the proposed cooperative control solution. Section 3.3 and Section 3.4 show open-loop and closed-loop simulation results. Discussions on the main results are given in Section 4. Finally, the conclusions are presented in Section 5.

## 2. Review of Pollution and Traffic Sensing Systems

In the last years, the interest in collecting data in cities has increased substantially. The current trend is to make a better use of public resources while increasing the quality of the services that citizens can access [21]. In particular, urban air quality sensing and traffic information are attracting a lot of research because of growing population in cities. In this section, a brief review about pollution and traffic sensing systems in cities is presented.

### 2.1. Pollution Sensing Systems

The basis for the consensus-based cooperative control is the pollution sensing system. Current sensors for pollution concentration measurement are based on different chemical of physical principles such as chemoresistance, solid electrolyte, absorption, etc. These sensors can measure different pollutants such as O_2_, O_3_, CO, carbon dioxide (CO_2_), mono-nitrogen oxides (NO_x_), PM, and volatile organic compounds (VOCs), in different range of sensitivity, selectivity and response time. Depending on the application, the sensor can be chosen with the required specific parameters e.g., in [22] an O_2_ with a sampling time of 1.6 s and NO_2_ with a sampling time of 41 s are selected.

Traditional approaches for pollution monitoring in urban or rural areas are based on networks of fixed stations for measuring air quality. Typically, these networks can provide detailed data but limited to the location of the stations. This makes that modelling approaches are usually used for obtaining representative pollution information in the area of interest [23].

The current challenges in the assessment of human exposure to pollution are how to make available reliable pollution data in cities, and how to handle large amounts of data from high resolution sensors and process it. Most recent methods propose the deployment of low-cost sensors that can provide emission information that allows the development of mitigation strategies [23]. In contrast with traditional approaches, the advances in sensor compactness, robustness, and wireless communications let sensor networks to be remotely managed, to easily transmit collected data and to report high spatial-resolution information in near-real time [24]. Data from these new pollution information systems enable a better pollution assessment and the development of new control strategies for pollution reduction [23].

### 2.2. Traffic Sensing Systems

The performance and safety of traffic control and management systems depend directly on traffic sensing systems. As in the case of pollution sensing systems, in the last decade there has been significant progress in the application of computer, sensing and communication technologies to traffic management and sensing systems [25]. Nowadays, there exist different technologies for traffic sensing that are suitable for several scenarios depending on the installation of the sensors, maintenance, performance, atmospheric conditions, etc. Some of them are: inductive loops, radio-frequency identification (RFID), microwave radar, acoustic, magnetic, ultrasonic and video image processor (VIP) [25]. Inductive loop detectors are commonly used for traffic sensing in cities and highways due to their characteristics: they are suitable for different weather conditions and different traffic volumes [26]. They also provide consistent and accurate measurements. Induction loops are also capable of measuring vehicle speed and vehicle classification, what make them appropriate and reliable for provide data to traffic control systems [27].

## 3. General Scenario and the Proposed Solution

**Figure 1:**
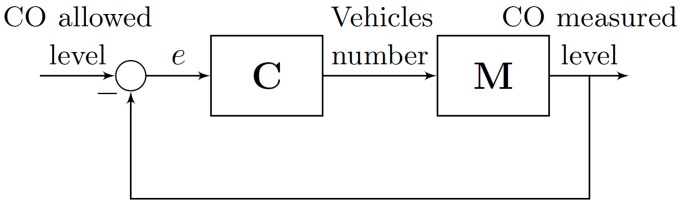
The schematic concept of a vehicle emission control system.

We have defined a general scenario based on a smart mobility application with the aim of improving the performance of urban traffic networks in urban regions of a city. The scenario is based on an emission control scheme proposed by Andò et al. [28]. These authors suggested the idea of a vehicles emission control scheme for traffic flow control as a possible solution to address the problem of urban air quality (see Figure 1). However, they do not provide specific solution for designing the emission controller. Authors proposed the number of allowed vehicles as the main control signal whereas the difference between the innocuous allowed CO level and the CO measured level is the input signal.

A new control scheme and a procedure that can be integrated into the Adaptive Traffic Control System (ATCS) of an urban area are proposed in this work [29]. The ATCS adjusts, in real time, signal timing plans based on the current traffic conditions, demand, system capacity, among other conditions. In the control scheme suggested in this work, we focused on designing the adaptive component Δu for traffic lights cycles U. The traffic lights cycles can vary on the basis of the following relationship $U_{0}=U_{0}+U_{0}\Delta u$, where $U_{0}$ is the value set by other components of the ATCS or by traffic engineers.

**Figure 2:**
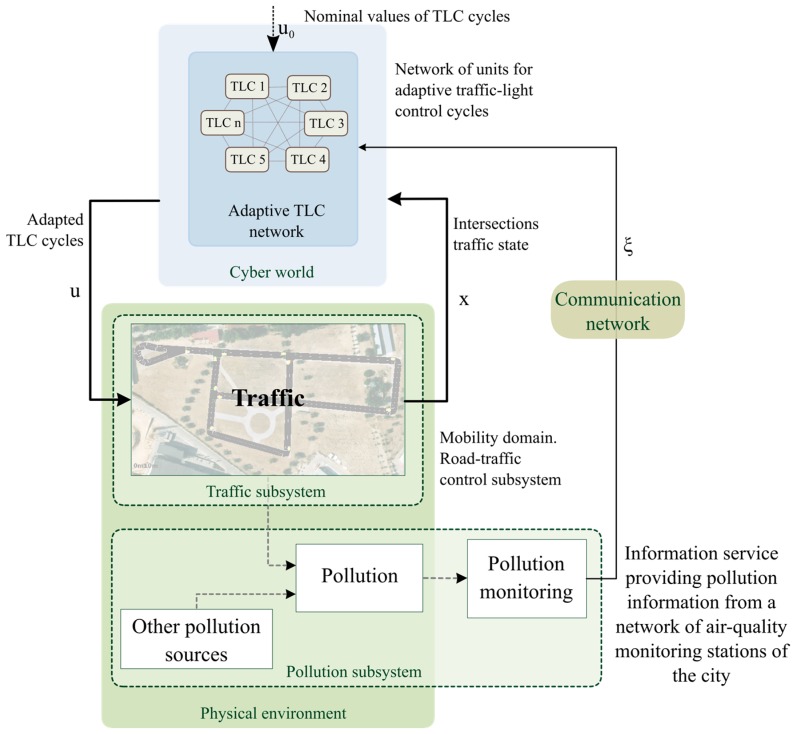
Diagram of the general scenario.

Figure 2 depicts a general diagram of the scenario and the strategy proposed in this work. Interconnected traffic lights control (*TLC*) network adapts the traffic-light cycles based on air pollution data provided by the city information service and local traffic measurements. The air pollution information service provides data (ξ) from the city pollution sensing system. Moreover, traffic information (x) is given by the vehicle detection sensor placed at each intersection of the urban traffic network. The traffic lights cycles (U) are updated by *TLC* units which make decisions based on a consensus variable (ε) derived from the consensus-based control method.

The following subsections address the modeling, control design, and simulation of the whole system.

### 3.1. Modeling

In order to study the dynamic behaviour and the interaction between the different components of the scenario, Discrete Event Systems Specification (DEVS) formalism is selected as a framework for SoS modeling and simulation. This formalism can be used for modeling and simulating complex dynamical systems and their interactions [20]. The DEVS modeling-based approach enables specification of basic models and how they are connected together. These basic models, known as atomic models, are modular systems with inputs (through input ports), changing states, and outputs (through output ports) running in a time frame. The couplings between atomic models generate coupled models.

In this work we used Parallel DEVS formalism (PDEVS) which solves collisions between internal and external transitions allowing all components to be activated and to send their output to other components. A basic PDEVS model can be represented as:

(1) $$M=\langle X_{M},Y_{M},S,\delta_{ext},\delta_{int},\delta_{con},\lambda ,t_{a}\rangle$$

where,

$X_{M}=\left\{\left(p,v\right)|p\in IPorts,\;v\in X_{p}\right\}$ is the set of input ports (*p*) and values (*v*)
$Y_{M}=\left\{\left(p,v\right)|p\in OPorts,\;v\in Y_{p}\right\}$ is the set of output ports (*p*) and values (*v*)
S is the set of states
$\delta_{int}:S\to S$ is the internal transition function
$\delta_{ext}:Q\times X_{M}^{b}\to S$ is the external transition function
$\delta_{con}:Q\times X_{M}^{b}\to S$ is the confluent transition function
$Q=\left\{\left(s,e\right)|s\in S,\;e\in \left[0,t_{a}\right]\right\}$ is the total state set and *e* is the time since the previous transition
λ:S→Y is the output function
$t_{a}:S\to R_{0}^{+}\cup \infty$ is the time advance function

Basic PDEVS models have a *bag* of inputs. A *bag* is a set with possible multiple occurrences of its elements e.g., {a,b,a,c} [30]. PDEVS also introduces the confluent transition function ($\delta_{con}$), which decides the next state in case of *collision* between external and internal events ($e=t_{a}\left(s\right)$) without the need for a priority scheme. This option provides a complete control over the *collision* behavior. Instead of serializing model behavior through the *select function* at the coupled-model level, PDEVS leaves this decision to the individual component. After modeling, it is necessary to conduct simulation studies. DEVS simulation methods generate the corresponding behaviors of each model, which are trajectories that can even reach illegal states by following execution protocols. This modeling paradigm serves to represent each subsystem as an atomic model that can be coupled with other model, forming coupled models.

**Figure 3:**
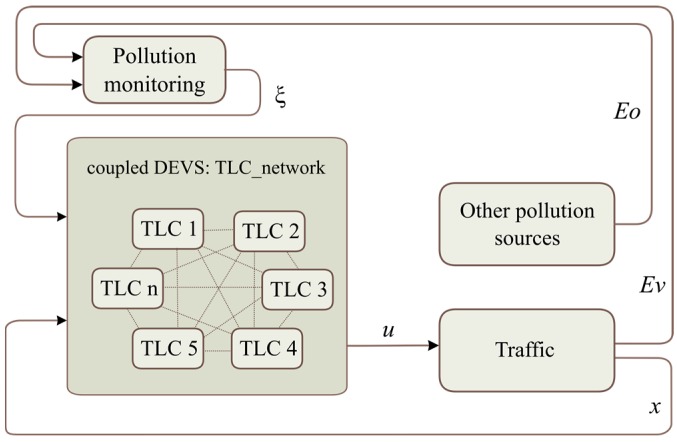
Structure of DEVS models.

This section addresses the representation of the system based on a conceptual model and the data flows between subsystems. Every system is represented as an atomic discrete system. The coupling of atomic models forms a coupled model. Figure 3 depicts the structure of a DEVS model of the general scenario and how the subsystems are connected.

In the above model, the following systems are defined as atomic models:

- Traffic-light control unit (*TLC*)
- Pollution-monitoring service
- Traffic system (i.e., road network, vehicles, traffic lights, etc.)
- Other pollution sources

The network of interconnected *TLCs* shares information (consensus variable, $\varepsilon_{i}$) to achieve a consensus between each other. This network is modeled as a coupled model. Each *TLC* is connected with a *Traffic* system to adapt the cycle length ($u_{i}$) of the corresponding traffic lights. The output of the *Traffic* subsystem is the traffic information (i.e., traffic flow at every intersection, $x_{i}$) and the pollution data from urban road traffic (*Ev*). The system called “*Other pollution sources*” emulated the pollution contribution of other urban subsystems (e.g., building heating systems, other transportation systems, etc., *Eo*). The *Pollution monitoring system* is represented by the urban pollution-measurement system that brings information on air quality to the *TLC network* (ξ). Data flows illustrated in Figure 2 and Figure 3 are summarized in Table 1.

### 3.2. Consensus-Based Cooperative Control Design

Consensus algorithms are thought to be distributed algorithms that assume only neighbor-to-neighbor interactions between subsystems. Subsystems update their information states based on their neighbors. The aim is to design a control strategy for updating the information states of all of the subsystems in the network, so that they converge to a common value.

The main rationale of a consensus-based procedure is to impose similar dynamics on the information states of each subsystem of the network [9]. Depending on the typology of the network for connecting subsystems, two different approaches can be applied. Firstly, if the network allows continuous communication, then the information state is modeled using differential equations. Secondly, if the data are communicated through discrete packets, the information state is represented by difference equations.

The topology for interactions in a network of agents is represented using a graph G=(V,E), where V={1,2,…,n} is a finite none-empty node set and E⊆V×V is an edge set of ordered nodal pairs, called *edge.* The edge (*i*, *j*) in the edge set of a graph denotes that node *j* can obtain information from node *i*. The neighbors of a node *i* are denoted by $N_{i}=\left\{j\in V:\left(i,j\right)\in E\right\}$. An iterative form of the consensus algorithm to reach an agreement in relation to the state of *n* integrator nodes with dynamics ${\dot{x}}_{i}=u_{i}$ can be represented in discrete time as follows:

(2) $$x_{i}\left(k+1\right)=x_{i}\left(k\right)+\lambda \underset{j\in N_{i}}{\sum }a_{ij}\left(x_{j}\left(k\right)-x_{i}\left(k\right)\right),\;k\in \mathbb{N}$$

where, $a_{ij}$ is the entry in row *i* and column *j* of the adjacency matrix $A_{n}\in {\mathbb{R}}^{n\times n}$ associated with G, and $x_{i}$ is the information state of the *i*-th system. A consequence of this equation is that the information state $x_{i}\left(k\right)$ of the system *i* is driven toward the information states of its neighbors. The consensus is achieved if, for all $x_{i}\left(0\right)$ and for all i,j=1,…,n, $\left|x_{i}\left(k\right)-x_{j}\left(k\right)\right|\to 0$, as k→∞.

The procedure to apply the consensus-based control and the results are then presented. The goal of the control system is to achieve a consensus between the intersections related to an estimation of local emissions (consensus state variable) by adapting local traffic-light cycles. The consensus-based decision-making takes into consideration the air-pollution state, provided by the air pollution information service, and the local traffic state. Specifically, a discrete consensus-based control algorithm is applied to control the coordination of the *TLC network*. The main steps for designing the control system are described in the following subsections.

#### 3.2.1. System Dynamics

A consensus variable that represents the system dynamics should be defined. Each *TLC* uses local traffic and pollution sensing information from a pollution information service. Therefore, an estimation of the pollutant concentration at each intersection as the consensus state variable is taken. The dynamics of each intersection is the same and it is assumed to behave as a local linear system. The discrete dynamic model of the node, that includes traffic information and pollution sensing data, is defined by the following recursive equation:

(3) $$\varepsilon_{i}\left(k+1\right)=\varepsilon_{i}\left(k\right)+\alpha_{i}\xi \left(k-n\right)+\beta x_{i}\left(k-m\right)+\gamma \Delta u_{i}$$

where,

- $\varepsilon_{i}\left(k\right)$ [gNO_x_/m^3^] is the state of the system i at instant k. The state of the system is related to the pollution levels and the traffic state at each intersection. The pollutant considered in this study is NO_x_ (mononitrogen oxides).
- ξ(k−n) [gNO_x_/m^3^] is a system input. It contains the pollution information provided to the *TLCs* at instant k−n where n is the delay between pollution production and pollution information reception by the *TLCs*.
- $\alpha_{i}$ is a dimensionless parameter that represents the contribution of intersection i to overall city pollution. It is computed on the basis of the maximum occupancy of intersection i over the total maximum occupancy.
- $x_{i}\left(k-m\right)$ [veh] is a system input. It contains the summation of vehicle queues at every approach of the intersection controlled by *TLC_i_*. m represents the delay between traffic queue measurement and traffic information reception at *TLC_i_*.
- β [gNO_x_/veh/m^3^] is the pollutant emissions of a given traffic queue at an intersection. $\beta =\frac{q\cdot F}{10^{3}\cdot \Delta t}$, where q is the emission factor of the pollutant (gNO_x_/veh/km), F is the dispersion factor of the pollutants (s/m^2^) and Δt is the simulation step [31].
- $\Delta u_{i}$ [% T.L. cycle] is the consensus-based cooperative control output of *TLC_i_*. The control signal is a percentage change of the cycle length of the traffic lights with respect to the initial cycle length in a limited range. This component is defined later (control law design).
- γ [gNO_x_/m^3^/% T.L. cycle] represents the influence of the pollutant emissions on traffic-light timing. $\gamma =\beta \gamma^{\prime }$, where $\gamma^{\prime }$ [veh/% T.L. cycle] is a parameter that represents the queue length with respect to changes in traffic-light cycle lengths. Therefore there is a proportional relationship between γ and $\gamma^{\prime }$.

#### 3.2.2. Design of a Consensus-Based Control Strategy

The control strategy adopted for each *TLC* (nodes of the network) makes use of pollution and traffic information as well as information from neighbors. Taking into account the dynamic model considered in (2) and applying the consensus control described in (1), the control strategy is represented by:

(4) $$\Delta u_{i}\left(k\right)=-\frac{1}{\gamma }\left(\alpha_{i}\xi \left(k-n\right)+\beta x_{i}\left(k-m\right)+\lambda \underset{j\in N_{i}}{\sum }a_{ij}\left(\varepsilon_{i}-\varepsilon_{j}\right)\right)$$

where,

- λ is a parameter that refers to system stability. If $\lambda \in \left(0,\theta^{-1}\right]$, where θ is the maximum degree of the graph, consensus convergence is guaranteed in a connected graph [32].
- $a_{ij}$ is the corresponding value of the adjacency matrix.

This control law contains three components, shown in Table 2.

In order to avoid large dissimilarities with the original traffic-light cycle lengths, the values of the control input $\Delta u_{i}$ are constrained to a variation of ±50% over the initial value.

### 3.3. Open-Loop Simulation for Test Scenario: Pollution and Traffic-Based Control Are Switched Off

**Figure 4:**
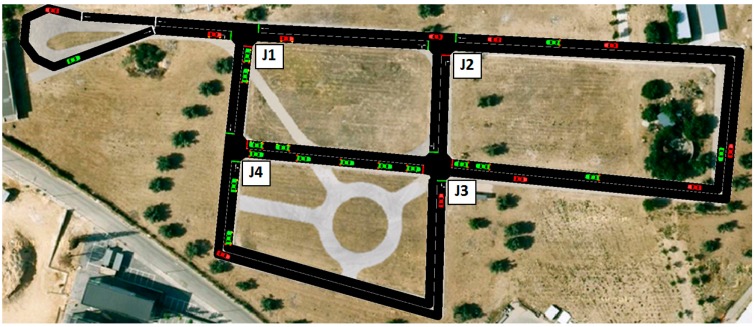
Simulated road network.

The scenario is based on an urban-like road network depicted in Figure 4. In order to simplify the description of the proposed solution, we considered a test scenario composed of four signalized traffic intersections (junctions J1, J2, J3 and J4) and interconnected *TLCs*. Vehicles are moving on the road network, following predefined routes randomly generated for imitating urban traffic. The vehicles included in the scenario are of the same type and use the same car-following model which is a version of the model defined by Krauß et al. [33]. In this version, different deceleration capabilities of the vehicles are handled without violating safety (the original model allowed for collisions in this case), and the formula for safe velocity is adapted to maintain safety when using the current Euler-position update rule. The parameters defined for vehicles and the car-following model are shown in Table 3.

The number of trips is defined by the repetition rate (number of trips per second). The repetition rate is calculated randomly by using a normal distribution with a mean of 5 and standard deviation of 0.25. Routes were generated, ensuring a minimum straight-line distance between the start and the end edges of a 170-m trip.

**Figure 5:**
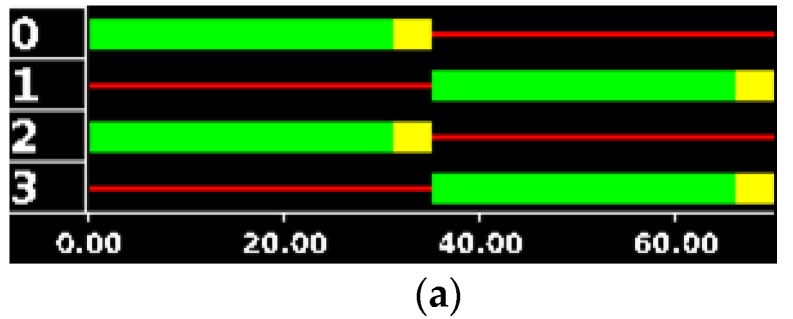

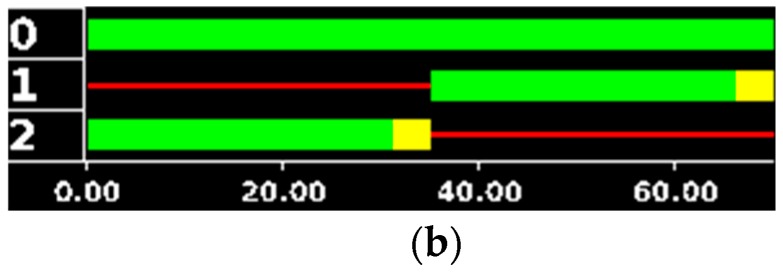
Initial timing of J3 (**a**), initial timing of J1, J2 and J4 (**b**).

In this scenario, *TLCs* work with a fixed timing $u_{0i}$ ($\Delta u_{i}=0$), because there is no control system running. This initial timing ($u_{0i}$) is shown in Figure 5. The simulation ran 2 h (7200 s) with a simulation step size of 1 s.

We used the SUMO simulator [34] for emulating the urban traffic network. This is a microscopic traffic simulator which enables interaction with an external application through an interface (TraCI: Traffic Control Interface). This simulator feeds traffic information from the traffic detectors, taking raw-pollution data of vehicles and traffic-light actions. TraCI4Matlab [35] is a library of functions for interaction with SUMO from the Matlab command line. It was used for linking SUMO simulator to Matlab models.

**Figure 6:**
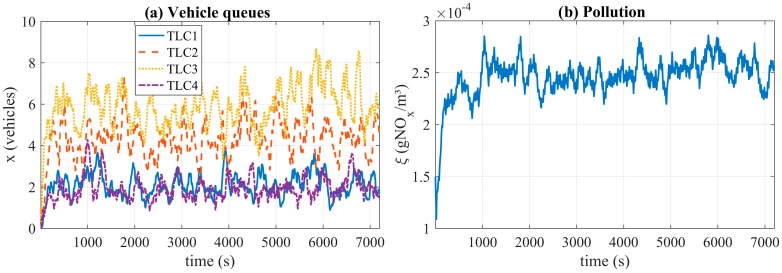
Vehicle queues (**a**) and pollution behavior (**b**) in an open-loop simulation.

Figure 6a shows the traffic queues obtained at all junctions. The plotted data corresponds to the average vehicle queues at every approach to each junction in a time window of 20 s. Figure 6b shows the average value of NO_x_ emissions of the whole scenario, also in a time window of 20 s. As can be noted, there are no vehicles in the scenario at the beginning of the simulation. Nonetheless, after 100 s from the start of the simulation, the vehicle numbers stabilize.

### 3.4. Consensus-Based Control Applied to the Test Scenario

**Figure 7:**
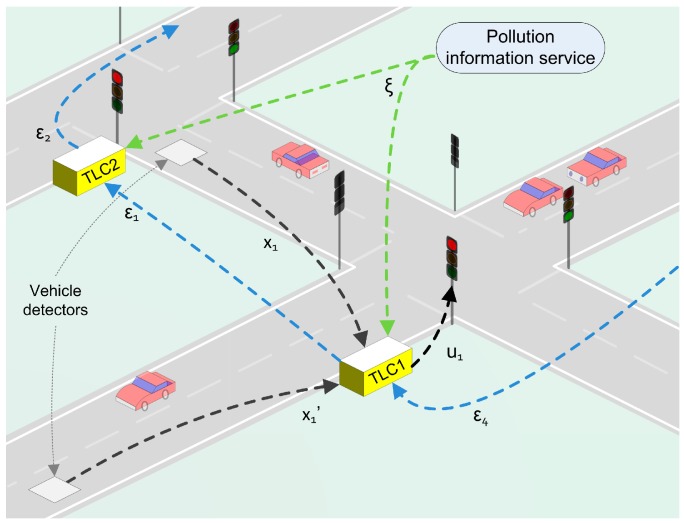
*TLCs* interaction scheme.

In this subsection, the simulation of the cooperative control system in the test scenario is described. The scenario is composed of four interconnected *TLCs*. The system components and the interaction between them are represented in Figure 7.

**Figure 8:**
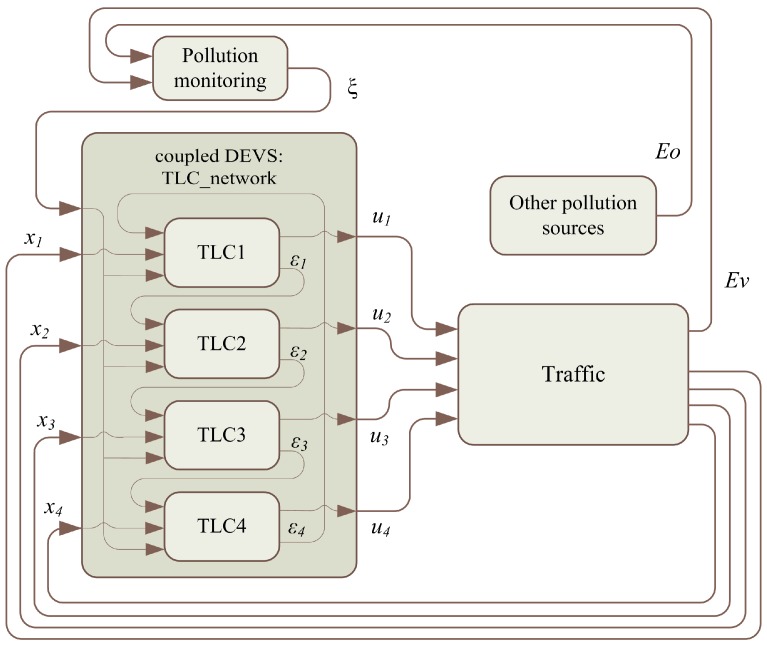
Schematic DEVS models for the test scenario.

The structure of DEVS model for carrying out the study is depicted in Figure 8. MatlabDEVS Toolbox [36] is used for the implementation of the DEVS models and the DEVS simulation. This toolbox has the necessary functions and scripts related with PDEVS formalism, making use of object-oriented language. This toolbox also permits us to run the PDEVS simulator and hybrid simulations by using continuous variables within atomic models.

**Figure 9:**
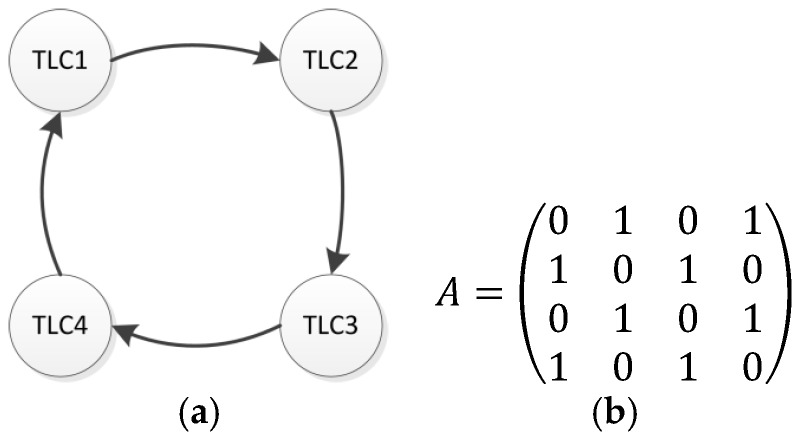
Network communication topology (**a**) and associated adjacency matrix (**b**).

Figure 9 describes the network topology and the associated adjacency matrix. The adjacency matrix represents the connections of the nodes with their neighbors. The communication topology is defined by a directed cycle graph composed of four nodes for this specific test scenario. This communication topology is a key issue for emulating the system behavior. We can extract the adjacency matrix from this representation. If a communication link exists the corresponding value is $a_{ij}=1$, otherwise $a_{ij}=0$.

For testing the whole system, the scenario was run during 7200 s (2 h) and the control system for *TLCs* starts at second 100. The following parameters of the models were defined for the simulation of the control system:

- The pollutant considered in this work was NO_x_. The NO_x_ produced by vehicles was taken from the HBEFA [37], assuming the vehicles were passenger cars built between 2005 and 2015 (0.35 gNO_x_/veh/km).
- The DEVS model of “*Other pollution sources*” produces pollution information every 5 s from statistical data of a common urban area, without taking into account the traffic contribution to the pollution. It generates random numbers from the normal distribution with mean: μ_Eo_ = 30.36 µgNO_x_/m^3^ and standard deviation σ_Eo_ = 10.48 µgNO_x_/m^3^.
- The DEVS model “*Pollution monitoring*” filters the input data by using a moving average filter with a window size of 100 s and outputs a value every 10 s.
- The execution period of *TLC* DEVS model is 1 s. It filters the input traffic data using a moving average filter with a window size of 100 s.
- The value of λ was set to 0.15 to guarantee consensus stability (λ∈(0,1/θ]).
- At each *TLC*, a threshold value of variation of 1% was defined for $\Delta u_{i}$, in order not to send continuous new $u_{i}$ values to the traffic lights when the variation of $u_{i}$ is minimal.
- The value of $\gamma^{\prime }$ was estimated by applying a linear regression with values obtained via simulation. The adopted value was 12.68 [veh/% T.L. cycle].

Each *TLC* calculates the new values of traffic queues (based on vehicles detector sensors placed at each intersection), the consensus variable $\varepsilon_{i}$ and the control signal $\Delta u_{i}$. After that, the cycle length of each traffic light is calculated and updated as $u_{i}=u_{0i}+u_{0i}\cdot \Delta u_{i}$, where $u_{0i}$ represents its initial value. Finally, the new value of $u_{i}$ is sent to the corresponding *TLC*, in order to modify the cycle length.

**Figure 10:**
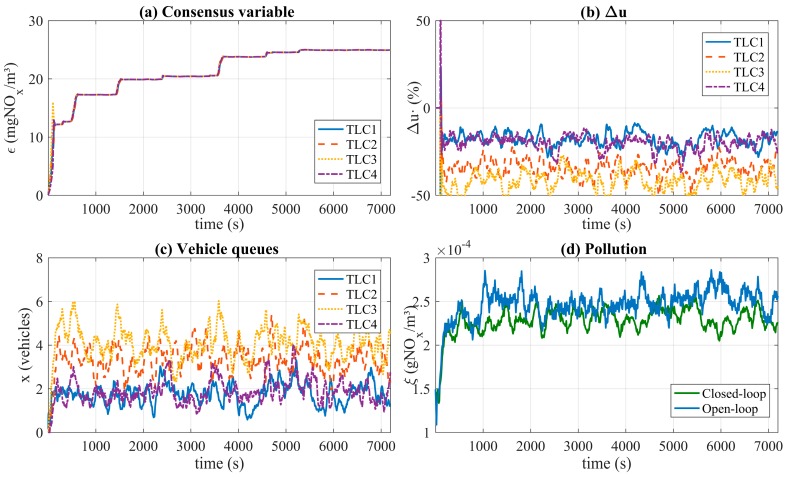
Close-loop simulation results: (**a**) Consensus variable (ε); (**b**) Control input (Δu); (**c**) Vehicle queues (x); (**d**) Pollution level (ξ) (closed-loop and open-loop).

Once the cooperative control is activated at second 100, each *TLC* begins to exchange information with the neighbors and calculates the control input $\Delta u_{i}$. Figure 10 shows the simulation results of the interconnected systems. Figure 10a shows the evolution of the consensus variable of each *TLC* when the control is activated. The consensus variable achieves the same values after 40 s of execution time. Figure 10c shows the amount of vehicles in each intersection (x) while Figure 10d reflects the total pollution level (ξ) in both cases open-loop and closed-loop control. The results depicted in this figure show a reduction in the total pollution level in comparison to the study presented in Section 3.3 without a control mechanism (i.e., open loop).

## 4. Results and Discussion

The behaviour of the system is highly dependent of traffic conditions, therefore 50 runs for simulating the scenario of both open loop (*TLCs* work with a fixed timing or $\Delta u_{i}=0$) and closed-loop were performed to carry the comparison. In every new simulation random vehicle routes were generated.

In order to assess the performance of the control system, two key performance indicators (KPIs) were defined, i.e., the mean absolute value of vehicle queues at all intersections and the global pollution during the simulation time (2 h). Both are selected because they serve to evaluate the actual behaviour of the consensus-based control system. The KPIs values are shown in Table 4.

In the case of vehicle queues, the average value for all simulations was 13.48 for open-loop and 12.04 for closed-loop, representing an improvement of 10.70%. Table 4 shows smaller KPIs for closed-loop strategy than open-loop ones. It also demonstrates that vehicle queues are therefore decreased by applying the consensus-based cooperative control system for the urban traffic network. Accordingly, the effect of balancing consensus variables in every *TLC* produces a global reduction of vehicle queues. As shown in Figure 10b,c, the control system implements a greater decrease in the timing of intersections with larger numbers of vehicles. This makes the number of vehicles at these intersections smaller compared to the case of the open-loop system.

Regarding the key performance indicator related to global pollution, there is a small reduction when the control system is activated. In the case of the mean value of this KPI, the difference between the close-loop and open-loop performance is not as significant as in the case of the KPI related to vehicle queues at all intersections. However, despite the presence of traffic pollution and disturbances in the pollution produced by other sources, we consider that the performance of control system is appropriate since a positive effect is achieved because global pollution is slightly reduced.

## 5. Conclusions

In this work a cooperative control approach for a smart city environment based on pollution sensing and traffic information is presented. By applying a System-of-Systems engineering design paradigm, a traffic control subsystem uses information from the city pollution sensing system for adjusting the cycle length of the traffic lights. Furthermore, a consensus-based control method was developed to address specific problems related with traffic congestion and air pollution. Discrete event system specification was used to represent and simulate the whole system. This modelling paradigm serves to deal with interoperability of heterogeneous systems.

The proposed method is evaluated in a defined scenario. The study demonstrates that the proposed method is a powerful strategy to deal simultaneously with different sensing systems and for integrating information from pollution levels and traffic flows in urban traffic networks. The results are promising because the number of vehicles in queue decreased, while consensus state variable at each intersection tended towards a common value, demonstrating the validity of the proposed solution. Further research will be conducted to test the solution in larger and more complex scenario and to extend the simulation results to real-time platforms.

## Figures and Tables

**Table 1:** Description of data flows.

Variable	Description
*Ev*	Vehicle emissions monitoring
*Eo*	Other emissions
ξ	Area-wide air-quality information. Includes current pollution-status details for a given geographic area.
ε	Consensus variable that represents the *TLC* dynamics (see Section 3.2.1)
x	Processed traffic-detector data which allows derivation of traffic-flow variables (density, occupancy, flow measures, etc.). It can be represented as a vector that refers to the signal of each sensor.
u	Data flow contains the system configuration data for a traffic signal controller. It includes the parameters required to reconfigure its operations.

**Table 2:** Components of the control method

Component	Expression	Description
1	$\alpha_{i}\xi \left(k-n\right)$	Feed-forward action related to local pollution data.
2	$\beta x_{i}\left(k-m\right)$	Feed-forward action related to local traffic data.
3	$\lambda \underset{j\in N_{i}}{\sum }a_{ij}\left(\varepsilon_{i}-\varepsilon_{j}\right)$	Consensus-based control signal that makes use of information from the neighbor of each network node.

**Table 3:** Vehicle and car-following model parameters.

Variable	Value
Length (m)	5.00
Minimum gap (m)	2.50
Maximum speed (m/s)	55.56
Maximum acceleration (m/s^2^)	2.60
Maximum deceleration (m/s^2^)	4.50
Imperfection	0.50
Reaction time (s)	1.00
Person capacity	4

**Table 4:** KPIs for the evaluation of the control system.

KPI		Open-Loop	Closed-Loop	Differences Relative to Open-Loop
Vehicle queues 1. $\frac{1}{t_{f}-t_{s}}\int_{t_{s}}^{t_{f}}∥x∥dt$	*μ*	13.4815	12.0382	10.70%
	max	15.0661	13.6345	9.50%
Global pollution 2. $\frac{1}{t_{f}-t_{s}}\int_{t_{s}}^{t_{f}}∥\xi ∥dt$	*μ*	2.3879×10^−4^	2.3791×10^−4^	0.37%
	min	2.2732×10^−4^	2.1910×10^−4^	3.62%
